# Alternating-Offers Bargaining with Nash Bargaining Fairness Concerns

**DOI:** 10.3390/bs13020124

**Published:** 2023-02-01

**Authors:** Zhongwei Feng, Fangning Li, Chunqiao Tan

**Affiliations:** 1 School of Business, Henan Polytechnic University, Jiaozuo 454000, China; 2 School of Business, Nanjing Audit University, Nanjing 211815, China

**Keywords:** Rubinstein alternating-offers bargaining game, subgame perfect equilibrium, fairness concerns, Nash bargaining solution, bilateral monopoly

## Abstract

The Rubinstein alternating-offers bargaining game is reconsidered, where players show fairness concerns and their fairness references are characterized by the Nash bargaining solution. The objective of this paper is to explore the impact of fairness concerns in the alternating-offer bargaining game. Alternating-offer bargaining with fairness concerns is developed. We construct a subgame perfect equilibrium and show its uniqueness. Then, it is shown that players’ payoffs in the subgame perfect equilibrium are positively related to their own fairness concern coefficient and bargaining power and negatively to the opponents’ fairness concern coefficient. Moreover, it is shown that the limited equilibrium partition depends on the ratio of discount rates of the two players when the time lapse between two offers goes to zero. Finally, the proposed model is applied to the bilateral monopoly market of professional basketball players, and some properties of equilibrium price are shown. Our result provides the implication that players should carefully weigh their own fairness concerns, bargaining power and fairness concerns of their opponents, and then make proposals, rather than simply follow the suggestion that the proposal at the current stage is higher than that at the past stages.

## 1. Introduction

Bargaining plays an important role in economics. This is partly since bargaining between and among firms and individuals has an effect on many aspects of economic activity and partly because bargaining occupies a critical place in economic theory. Bargaining is the case of economic interaction where the market only plays a role in setting the bounds of discussion, and the bargaining outcomes are determined by the strategic interaction of the players. In particular, sufficient information about bargaining, including the attributes of the players and the structure of the bargaining problem faced by the players, allows the range of indeterminacy to be narrowed or eliminated [1]. On the other hand, making offers and counteroffers plays an important role in many real-life negotiations, that is, an attractive procedure of bargaining is that both players take turns to make proposals to each other until an agreement is reached [2]. These views were illustrated by the alternating-offers bargaining game proposed by Rubinstein [3], which embodies a detailed description of a bargaining procedure. When players bargain over a pie, a basic source of the cost incurred by a player comes from the following facts that bargaining is time-consuming and time is valuable to the player. The Rubinstein bargaining model is a formal exploration of the role of the players’ discount rates in a time-consuming, offer–counteroffer process.

Different from the Nash bargaining model (i.e., the axiomatic approach) that tries to predict the equilibrium solution to the negotiation issue, Rubinstein’s bargaining, as a strategic approach, exactly describes the negotiation process in detail [4]. In the strategic approach, the bargaining procedure is explicitly characterized as a strategic game that is specified fully [5]. Rubinstein shows an agreement that arises from equilibria and predicts the unique agreement, which adds a dimension to the bargaining theory [6]. Rubinstein’s bargaining has a great deal of intuitive appeal because making offers and counteroffers refers to the heart in numerous real-life bargaining situations [7].

Although Rubinstein’s bargaining is convenient analytically, the utility specification in this model cannot explain fully the behavior preferences that are typically observed in some real bargaining situations. Thus, numerous scholars consider Rubinstein’s bargaining with behavior preferences, such as loss aversion [8,9,10], risk aversion [11,12], altruistic and spiteful preferences [13,14] and reference-dependent preferences [15,16]. Abundant experimental works show that a player often rejects an offer perceived as unfair, instead of maximizing the monetary payoff [17,18,19,20]. In particular, Bolton shows that a party is motivated by the absolute monetary payoff of himself or herself as well as the relative size of this monetary payoff compared with the payoff of the others [21]. The approach introduced by Bolton explains numerous experimental findings on the bargaining game, but it cannot explain why players in some situations are willing to pay for fair treatment [21]. For example, in 2007, Langsha group, which is the largest sock manufacturer in China, gave up cooperation with Wal-Mart, since the profit allocation for the Langsha group was unfair. Another example is that in 2010, Xuzhou Wanji Trading of China terminated transactions with P&G, since the latter grabbed profits too much. These imply that players’ fairness preferences impact whether players cooperate. That is, players’ fairness preferences receive attention in real bargaining situations. Thus, a natural extension to the classical Rubinstein bargaining is to incorporate fairness preferences.

To avoid latent misunderstandings, it is stressed that fairness is not outright altruism. An altruistic player may prefer to give away some payoff of himself or herself to another one. It is not necessarily case for a player with fairness preferences. A player with fairness preferences may in general be more prone to a higher payoff, even though this payoff difference would be obtained by other players [19,22]. For instance, there is usually a strict conflict of interest between players with fairness preferences in real bargaining. Nevertheless, for a player with fairness preferences, the loss of a share below the fair reference level creates a higher disutility than an equal-sized loss of a share above this fair reference level, which is different from a standard utility specification [22].

Although different fairness models are proposed by researchers [18,19,21], the fairness model introduced by Fehr and Schmidt [23], which is called the ‘F-S model’, incorporates more general equity into the utility functions of players. In the F-S model, with two players, the player’s payoff is regarded as the opponent’s fairness reference point. In some cases, this assumption may be reasonable, while in others it has its limitations. For example, a player with less bargaining power may not expect the fairness reference point. That is, there is a major issue in the F-S model that the exogenous fairness concern parameters cannot capture power and contribution endogenously and affect the fairness perception. To address this issue, we adopt the version of Du et al. that incorporates bargaining power to characterize players’ fairness concerns [24]. In this version, the asymmetric Nash bargaining solution is regarded as a fairness reference, since the axioms (i.e., Pareto optimality, invariance, symmetry and independence of irrelevant alternatives) that are used to describe the Nash bargaining solution define fairness [3,25,26]. The fairness reference requires that a player has to consider the opponent’s payoff in pursuing payoffs. On the other hand, the gain below the farness reference reduces the utility and the sensitivity of the gap between an outcome and the fairness reference point is characterized by the fairness concern parameter.

However, the significance of fairness preferences has received relatively little attention in the bargaining problem, with the exception of bargaining experiments [20,21,22,23,24,25,26,27,28,29]. For example, Guha argued that an interior bargaining solution would occur for one-sided inequality aversion when the discount factor tends to one [30]. Cao et al. examined the impact of inequality aversion on bilateral ultimatum bargaining with incomplete information [31]. Galeotti et al. explored how the bargaining outcome changes with changes in the trade-off between efficiency and equality [32]. Ma et al. investigated the impact of dual fairness concerns on the sequential bargaining game [33]. The above literature cannot provide a formal answer to the question “why players in some situations are willing to pay for fair treatment?” [17,19]. To answer the above question, Ewerhart modified the F-S model to stress fairness where a common agreement reached by the two players is regarded as their own reference level and adopted this modification model to study the effect of fairness on the Rubinstein bargaining game [34]. Kohler and Schlag adopted the F-S model to study the impact of inequality aversion on the Rubinstein bargaining game under the assumption that the fairness reference level of a player is identical to the payoff of the opponent [35]. Nevertheless, the fairness reference points for the above two works may not be expected by players with low bargaining power, since such reference points cannot capture endogenously bargaining power. Different from the above-mentioned studies, our work models the Rubinstein bargaining with fairness preferences under the assumption that the Nash bargaining solution is regarded as the fairness reference of each player.

Without loss of generality, a player’s bargaining power is given exogenously. That is, it is assumed that players settle with their relative positions given. For instance, the bargaining power of the union can be determined by price-cost margins, wage premia, level of employment and revenue of the Belgian industry [1]. In fact, bargaining power in many applications is assumed to be exogenous [36,37,38,39]. On the other hand, the bargaining power of the union is identified with its ability to influence the wage [40]. That is, in wage bargaining, a firm can determine the union’s bargaining power by the ability to influence the wage. In many real wage bargainings, many employment relationships are not spot market transactions in which workers and firms interact only once, but long-term relations that amplify the importance of fairness concerns. Thus, a strictly selfish individual has incentive to mimic a fair-concerned worker in a long-term employment relationship and to exert high-level effort when offered a lower wage, since it can guarantee a higher wage in the future [41]. In contrast, shirking shows that a worker is egoistic. It is impossible that firms pay higher wages to selfish individuals, once firms have proven that these workers don’t reciprocate generous treatment with high effort [41]. In such situations, it is likely that firms and workers report the real levels of their fairness concerns. Thus, in this paper, it is assumed that the fairness concern coefficients are common knowledge.

In this paper, we adopt the fairness model introduced by Du et al. [24] to characterize players’ fairness concerns and reconsider the Rubinstein alternating-offers bargaining game, where players care about fairness and the Nash bargaining solution is regarded as the fairness reference. For this bargaining game, we construct a subgame perfect equilibrium (SPE) and show its uniqueness. Then, some properties of SPE with respect to fairness concern coefficients and bargaining power are discussed. It is shown that higher fairness concerns can lead to a higher equilibrium share. Another interesting result is that higher bargaining power results in a higher equilibrium payoff. Finally, the proposed bargaining model is applied to the bilateral monopoly market, and the variation of equilibrium price with respect to fairness concern coefficients and the bargaining power is discussed.

Our work makes the following several contributions. Firstly, from the theoretical perspective, the present study contributes to the alternating-offer bargaining game literature by investigating players’ fairness preferences. This differs from Ewerhart [34] and Kohler and Schlag [35] in which Rubinstein’s bargaining with fairness preferences focuses on the reference level, regardless of a player’s bargaining power. In contrast, our work examines how players’ fairness preference as well as bargaining power affects SPE by the Nash bargaining fairness reference. This exploration enhances the understanding of the strategic bargaining game model with fairness preferences. Secondly, our work contributes to the Rubinstein bargaining game with reference-dependent preferences. Our study determines player’s fairness reference level based on the Nash bargaining solution, which reflects the impact of player’s bargaining power on the reference-dependent preferences. To the best of our knowledge, the impacts of bargaining power on the reference-dependent preferences have been not yet investigated in Rubinstein’s bargaining game.

The rest of this paper is organized as follows. Section 2 introduces the bargaining model with fairness concerns. The unique SPE is shown and some properties of the equilibrium are analyzed in Section 3. Section 4 presents an extended model with individual discount factors. In Section 5 an application to the bilateral monopoly market is given. In Section 6 a discussion is shown. And Section 7 gives conclusions as well as limitations.

## 2. Preliminaries

### 2.1. The Rubinstein Bargaining Model

Two players, 1 and 2, bargain over one unit of a pie. The set of all feasible divisions is denoted by

$$Z:=\{(z_{1},z_{2})|z_{1}+z_{2}=1,z_{1},z_{2}\geq 0\}.$$

Proposals are made at times *t* ∈ *T*: = {Δ, 2Δ,…}, where Δ > 0. Player 1 makes an offer *z* = (*z*_1_, *z*_2_) ∈ *Z* to player 2 at time *t* ∈ *T*_odd_: = {Δ, 3Δ,…}. If player 2 accepts this offer (*Y*), then the bargaining ends and player *i* (*i* = 1, 2) obtains the share *z_i_*. If player 2 rejects this offer (*N*), then player 2 makes a counteroffer to player 1 at time *t +* 1 ∈ *T*_even_: = {2Δ, 4Δ,…}. If this offer is accepted, then the bargaining ends. If player 1 rejects this offer (*N*), then player 1 makes another proposal to player 2 at *t +* 2 ∈ *T*_odd_: = {Δ, 3Δ,…}, and so on. If the bargaining continues forever, then the share of each player is zero.

The objective of each player is to maximize the expected discounted payoff with a discount factor *δ* < 1. Following the prior works (e.g., [42,43]), let *β* > 0 be a positive rate of time preference for a player, then δ=exp(−βΔ), where Δ is the length of time interval in the units of *β*. Since players maybe have the willingness to deviate from their initial action plan over time, the non-exponential discounting cannot ensure time consistency [44]. To address the issue of time consistency for players, exponential discounting is adopted. Such exponential discounting also extensively applies in economics [45].

Let $h^{t}:=(z^{1},z^{2},\ldots ,z^{t})$ denote the history of the bargaining game at time *t* ∈ *T*, where *z^s^* ∈ *Z* for all *s* ≤ *t*. Let $H^{t}=\Pi_{s=1}^{t}Z$ denote the set of all possible histories *h^t^* of the bargaining game at time *t* ∈ *T*. Let *F*, which is the set of all sequences of functions of *f* =${\left\{f^{t}\right\}}_{t=1}^{\infty }$, denote the set of strategies of player 1, where for *t* = 1: *f^t^* ∈Z, for *t* > 1 and *t* ∈ *T_odd_*, *f^t^*: *H ^t^*^−1^→ *Z* and for *t* ∈ *T_even_*, *f^t^*: *H^t^*→ {*Y*, *N*}. Similarly, let *G* denote the set of strategies of player 2, in other words, *G* is the set of all sequences of functions of *g* =${\left\{g^{t}\right\}}_{t=1}^{\infty }$, where for *t* ∈ *T_odd_*, *g^t^*: *H^t^* → {*Y*, *N*} and for *t* ∈ *T_even_*, *g^t^*: *H^t^*^−1^ → *Z*.

If an offer at time *t* is accepted, then an agreement path is denoted by (*h^t^*, *Y*), where *z^s^* ∈ *Z* for all *s* ≤ *t*. The set of all time *t* agreement paths is denoted by *A^t^*:={(*h^t^*, *Y*)|*h^t^* ∈ *H^t^* }. Let *A*: = ∪*_t_*_∈*T*_*A^t^*, then it contains all agreement paths. Similarly, a disagreement path is denoted by (*h^t^*, *N*), which means that the bargaining ends in breakdown at time *t*. We define *D^t^*: = {(*h^t^*, *N*)|*h^t^* ∈ *H^t^* } and *D*: = ∪*_t_*_∈*T*_*D^t^*. Finally, we define *H*^∞^: = {(*z*^1^, *z*^2^,…)|*z^t^* ∈ *Z* for all *t* ∈ *T*}, which implies that the element of *D^∞^* is referred to as infinite paths.

Note that (*f*, *g*) can determine a play of the bargaining game. Specifically, if it leads to an agreement on a proposal at time *t*, then the set of paths contains *t*−1 paths in *D* and one path in *A*. If it leads to perpetual disagreement, then the set of paths only contains a single path in *H^∞^*.

We define a function *τ_i_*: *A* (or *D*) → [0, 1], which reflects the share of the pie player *i* obtains in each finite path *A* (or *D*). Thus, for all *h^t^* ∈ *H^t^*, we have *τ_i_* (*h^t^*, *Y*)=$z_{i}^{t}$ and *τ_i_* (*h^t^*, *N*) = 0, where $z_{i}^{t}$ is player *i*’s share at time *t*.

### 2.2. Fairness Model

A fairness model was first introduced by Rabin [18]. However, the F-S model has received much attention, since this model successfully characterizes preferences for reciprocity concerns in many experiments. For the F-S model with two players, it is assumed that the payoff of the player is the fairness reference point of the other one. However, this assumption has its limitations when it comes to bargainers with less bargaining power, since the bargainer maybe not expect such a fairness reference point [24]. Thus, we adopt the fairness model introduced by Du et al. In this version, the Nash bargaining is just a psychological game for fairness perception that maybe not really happen but lie in players’ mind. That is, both players form fairness reference in mind first, which is based on the Nash bargaining game, and then play the alternating-offers bargaining game. Du et al. argued that the fairness reference consists of absolute fairness reference (i.e., the payoffs from the overall pie) and relative fairness reference (i.e., the payoffs from per unit pie). The latter is more persuasive than the former, because of a fair proportion regardless of the size of the pie. If the division of the pie is not realized by this rule, then unfairness occurred. Gain below the farness reference reduces the utility and the fairness concern coefficient *λ_i_* (*λ_i_* > 0) is used to characterize the sensitivity to this gap. Specifically, both players bargain over a pie, whose size is *π* (in this paper, *π* = 1). For a fairness-concerned player *i*, the utility is shown as follows.

$$\omega_{i}=\pi_{i}+\lambda_{i}(\pi_{i}-{\bar{\pi }}_{i})=(a_{i}+\lambda_{i}(a_{i}-r_{i}))\pi ,$$

where *λ_i_* > 0 is the fairness concern coefficient of player *i*. *π_i_* and *a_i_* are the realized material payoff and the proportion to the overall pie of player *i*, respectively, while ${\bar{\pi }}_{i}$ and *r_i_* are player *i*’s absolute fairness reference and relative fairness references, respectively. $\pi_{1}+\pi_{2}=\pi$, ${\bar{\pi }}_{1}+{\bar{\pi }}_{2}=\pi$, $a_{1}+a_{2}=1$ and $r_{1}+r_{2}=1$.

Since the relative fairness reference implies a fair proportion regardless of the size of the pie, we restrict ourselves to the relative fairness reference that is more persuasive. In this fairness model, the fairness reference implies that a player would not pursue self-interest without considering the opponent’s welfare. On the other hand, gain below the farness reference reduces the utility and the sensitivity to this gap is characterized by the fairness concern parameter *λ_i_* (*λ_i_* > 0). That is, Du et al. argued that the fairness concern parameter *λ_i_* captures the sensitivity to the gap between the gain and the fairness reference when the gain is less than the fairness reference.

This fairness model characterizes fairness concerns. The player *i*’s inherent properties can be characterized by the fairness concern coefficient *λ_i_* that does not depend on player *i*’s bargaining power. On the other hand, the fairness concern coefficient *λ_i_* can be used to characterize the fairness concern level of player *i*.

### 2.3. Fairness Reference Level

Since the relative fairness reference implies a fair proportion regardless of the size of the pie, we restrict ourselves to the relative fairness reference.

**Theorem** **1.** *The relative fairness reference points of players 1 and 2 satisfy, respectively*

$$r_{1}=\frac{\alpha (1+\lambda_{1})}{1+\alpha (\lambda_{1}-\lambda_{2})+\lambda_{2}}\;\mathrm{and}\;r_{2}=\frac{\left(1-\alpha )(1+\lambda_{2}\right)}{1+\alpha (\lambda_{1}-\lambda_{2})+\lambda_{2}},$$

*where α reflects the bargaining power of player 1 and 1 − α reflects the bargaining power of player 2.*

**Proof.**  To show relative fairness reference of player *i*, we focus on player *i*’s utility per unit pie. By Nash’s axiomatic definition [5,24,25], the Nash bargaining solution (*r*_1_, *r*_2_) is the partition (*a*_1_, *a*_2_) that satisfies the following model:

(1) $$\begin{array}{l} \max\;\psi ={\left(a_{1}+\lambda_{1}(a_{1}-r_{1})\right)}^{\alpha }{\left(a_{2}+\lambda_{2}(a_{2}-r_{2})\right)}^{1-\alpha }\\ s.t.\{\begin{array}{c} a_{1}+a_{2}=1\\ 0\leq a_{1},a_{2}\leq 1 \end{array} \end{array}$$

where 0 < *α* < 1 reflects the player 1′s bargaining power, while 1 − *α* is player 2′s bargaining power. *a_i_* + *λ_i_* (*a_i_* − *r_i_*) is player *i*’s utility per unit pie.

For simplicity, model (1) can be transformed into as follows:

(2) $$\max\;\ln\psi =\alpha \ln(a_{1}+\lambda_{1}(a_{1}-r_{1}))+(1-\alpha )\ln(1-a_{1}+\lambda_{2}(r_{1}-a_{1}))$$

Differentiating with respect to *a*_1_, we have

(3) $$\frac{d(\ln\psi )}{da_{1}}=\frac{\alpha (1+\lambda_{1})}{a_{1}+\lambda_{1}(a_{1}-r_{1})}-\frac{\left(1-\alpha )(1+\lambda_{2}\right)}{1-a_{1}+\lambda_{2}(r_{1}-a_{1})}$$

The second derivative of Equation (2) with respect to *a*_1_ is shown as follows:

$$\frac{d^{2}(\ln\psi )}{da_{1}{}^{2}}=-\frac{\alpha {\left(1+\lambda_{1}\right)}^{2}}{{\left(a_{1}+\lambda_{1}(a_{1}-r_{1})\right)}^{2}}-\frac{(1-\alpha ){\left(1+\lambda_{2}\right)}^{2}}{{\left(1-a_{1}+\lambda_{2}(r_{1}-a_{1})\right)}^{2}}<0,$$

which implies that lnψ is strictly concave in *a*_1_.

Thus, let Equation (3) be equal to zero, i.e.,

(4) $$\frac{\alpha (1+\lambda_{1})}{a_{1}+\lambda_{1}(a_{1}-r_{1})}-\frac{\left(1-\alpha )(1+\lambda_{2}\right)}{1-a_{1}+\lambda_{2}(r_{1}-a_{1})}=0$$

we can obtain a unique optimal solution.

On the other hand, if (*a*_1_, *a*_2_) is also the Nash bargaining solution of the model (1), then we have *a*_1_ = *r*_1_. Thus, the Nash bargaining solution is given as follows:

$$r_{1}=\frac{\alpha (1+\lambda_{1})}{1+\alpha (\lambda_{1}-\lambda_{2})+\lambda_{2}}\;\mathrm{and}\;r_{2}=1-r_{1}=\frac{\left(1-\alpha )(1+\lambda_{2}\right)}{1+\alpha (\lambda_{1}-\lambda_{2})+\lambda_{2}}.$$

These complete the proof of this theorem. □

### 2.4. Utility Functions and a SPE

Let an agreement path be denoted by (*h^t^*, *Y*) and a disagreement path be denoted by (*h^t^*, *N*) at time *t*. Then, player *i*’s utility for agreement paths is defined as

$$u_{i}(h^{t},Y,\lambda_{i},r_{i})=\omega_{i}(h^{t},\lambda_{i},r_{i})$$

According to Theorem 1, we have $r_{1}=\frac{\alpha (1+\lambda_{1})}{1+\alpha (\lambda_{1}-\lambda_{2})+\lambda_{2}}$ and $r_{2}=\frac{\left(1-\alpha )(1+\lambda_{2}\right)}{1+\alpha (\lambda_{1}-\lambda_{2})+\lambda_{2}}$.

Player *i*’s utility for disagreement paths is defined as

$$u_{i}(h^{t},N,\lambda_{i},r_{i})=0$$

If the agreement cannot be reached forever, then the utility of each player is zero.

Let *U_i_*: *F* × *G* → *R* be the expected utility function and the strategy profile (*f*, *g*) ∈ *F* × *G* be played from the moment *t* ∈ *T*, where *t* is the moment up until that the history is known. Then (*f*|*h^t^*, *g*|*h^t^*) is played at moment *t* + 1 and *U_i_* (*f*|*h^t^*, *g*|*h^t^*) is defined as the expected utility of player *i* at time *t* if (*f*, *g*) ∈ *F* × *G* is played.

**Definition** **1.** *In the alternating-offers bargaining game with fairness concerns, the strategy profile (f, g) is a SPE if for every t ∈ T and h^t^ ∈ H^t^,*

$$U_{1}\;(f|h^{t},\;g|h^{t})\geq U_{1}\;(\tilde{f}|h^{t},\;g|h^{t})\;\mathrm{for}\;\mathrm{all}\;\tilde{f}\in F$$

*and*

$$U_{2}\;(f|h^{t},\;g|h^{t})\;\geq \;U_{2}\;(f\;|h^{t},\tilde{g}|h^{t})\;\mathrm{for}\;\mathrm{all}\tilde{g}\in G.$$

## 3. SPE Analysis for Alternating-Offers Bargaining Games with Fairness Concern

To construct a SPE, we consider the following properties: (i) No delay. Whenever a player makes a proposal, the equilibrium proposal made by the player is accepted by the opponent. (ii) Stationarity. In equilibrium, whenever a player makes a proposal, the player makes the same proposal. Given property (ii), let $x^{*}=(x_{1}^{*},x_{2}^{*})$ ∈ *Z* denote the equilibrium proposal made by player 1 at time *t* ∈ *T_odd_*, and let $y^{*}=(y_{1}^{*},y_{2}^{*})$ ∈ *Z* denote the equilibrium proposal made by player 2 at time *t* ∈ *T_even_*. Consider an arbitrary odd time *t* at which player 1 makes a proposal to player 2. According to properties (i) and (ii), player 2′s equilibrium payoff from rejecting any proposal satisfies $\delta (y_{2}^{*}+\lambda_{2}(y_{2}^{*}-r_{2}))$. This is because, by property (ii), player 2 makes a proposal y^*^ after rejecting any proposal, which is accepted by player 1 by property (i). Perfection requires that player 2 accepts any proposal *x* = (*x*_1_, *x*_2_) ∈*Z* made by player 1 such that $x_{2}+\lambda_{2}(x_{2}-r_{2})>\delta (y_{2}^{*}+\lambda_{2}(y_{2}^{*}-r_{2}))$ and rejects any proposal *x* = (*x*_1_, *x*_2_) ∈*Z* made by player 1 such that $x_{2}+\lambda_{2}(x_{2}-r_{2})<\delta (y_{2}^{*}+\lambda_{2}(y_{2}^{*}-r_{2}))$. Furthermore, by property (i), $x_{2}^{*}+\lambda_{2}(x_{2}^{*}-r_{2})\geq \delta (y_{2}^{*}+\lambda_{2}(y_{2}^{*}-r_{2}))$. However, there does not exist x^*^ such that $x_{2}^{*}+\lambda_{2}(x_{2}^{*}-r_{2})>\delta (y_{2}^{*}+\lambda_{2}(y_{2}^{*}-r_{2}))$; otherwise, the payoff of player 1 could be improved by making a proposal $x^{\prime }=(x_{1}^{\prime },x_{2}^{\prime })$ such that $x_{2}^{*}+\lambda_{2}(x_{2}^{*}-r_{2})>x_{2}^{\prime }+\lambda_{2}(x_{2}^{\prime }-r_{2})>\delta (y_{2}^{*}+\lambda_{2}(y_{2}^{*}-r_{2}))$. Thus,

(5) $$x_{2}^{*}+\lambda_{2}(x_{2}^{*}-r_{2})=\delta (y_{2}^{*}+\lambda_{2}(y_{2}^{*}-r_{2}))$$

Similarly, we have

(6) $$y_{1}^{*}+\lambda_{1}(y_{1}^{*}-r_{1})=\delta (x_{1}^{*}+\lambda_{1}(x_{1}^{*}-r_{1}))$$

at time *t* ∈ *T_even_*.

Combining Equations (5) and (6) with Theorem 1, we can obtain equilibrium proposals:

$$\begin{array}{l} x^{*}=(\frac{1}{1+\delta }-\frac{(1-\alpha )\lambda_{2}-\delta \alpha \lambda_{1}}{\left(1+\delta )(1+\alpha (\lambda_{1}-\lambda_{2})+\lambda_{2}\right)},\frac{\delta }{1+\delta }+\frac{(1-\alpha )\lambda_{2}-\delta \alpha \lambda_{1}}{\left(1+\delta )(1+\alpha (\lambda_{1}-\lambda_{2})+\lambda_{2}\right)})\\ y^{*}=(\frac{\delta }{1+\delta }+\frac{\alpha \lambda_{1}-(1-\alpha )\delta \lambda_{2}}{\left(1+\delta )(1+\alpha (\lambda_{1}-\lambda_{2})+\lambda_{2}\right)},\frac{1}{1+\delta }-\frac{\alpha \lambda_{1}-(1-\alpha )\delta \lambda_{2}}{\left(1+\delta )(1+\alpha (\lambda_{1}-\lambda_{2})+\lambda_{2}\right)}). \end{array}$$

Now, the strategy $\hat{f}$ for player 1 and the strategy $\hat{g}$ for player 2 are defined based on the proposals x*and y*, respectively. The definition of the strategy $\hat{f}$ is given as follows: player 1 makes the offer x* at any odd time *t*, then player 1 accepts an offer *y* = (*y*_1_, *y*_2_) ∈ *Z* if and only if $y_{1}+\lambda_{1}(y_{1}-r_{1})\geq \delta (x_{1}^{*}+\lambda_{1}(x_{1}^{*}-r_{1}))$ at any even time *t*. Similarly, we define the strategy $\hat{g}$: Player 2 always makes the proposal y* at any even time* t* and always accepts a proposal *x*= (*x*_1_, *x*_2_) ∈ *Z* made by player 1 at any odd time *t*, if and only if $x_{2}+\lambda_{2}(x_{2}-r_{2})\geq \delta (y_{2}^{*}+\lambda_{2}(y_{2}^{*}-r_{2}))$ .

**Theorem** **2.** *The strategy profile (* $\hat{f},\hat{g}$ *) is a SPE in the alternating-offers bargaining game with fairness concerns, then the equilibrium partition is*

$$x^{*}=(\frac{1}{1+\delta }-\frac{(1-\alpha )\lambda_{2}-\delta \alpha \lambda_{1}}{\left(1+\delta )(1+\alpha (\lambda_{1}-\lambda_{2})+\lambda_{2}\right)},\frac{\delta }{1+\delta }+\frac{(1-\alpha )\lambda_{2}-\delta \alpha \lambda_{1}}{\left(1+\delta )(1+\alpha (\lambda_{1}-\lambda_{2})+\lambda_{2}\right)}).$$

**Proof.**  Given player 2′s strategy $\hat{g}$, we show that the strategy $\hat{f}$ is optimal at any time. Consider the case that *t* is odd. If player 1 plays $\hat{f}$, then the share of the pie that player 1 obtains is $x_{1}^{*}$.Consider any strategy *z* for player 1 at time *t*, if *z*_1_ = $x_{1}^{*}$, then player 1 follows strategy $\hat{f}$ and player 2 accepts it. If *z*_1_ < $x_{1}^{*}$, which implies that *z*_2_ > $x_{2}^{*}$, then this offer is accepted by player 2. Since $z_{1}+\lambda_{1}(z_{1}-r_{1})<x_{1}^{*}+\lambda_{1}(x_{1}^{*}-r_{1})$, it implies that *z* is not optimal. If *z*_1_ > $x_{1}^{*}$, then *z*_2_ < $x_{2}^{*}$. Player 2 rejects this offer and makes an offer y* at time *t* + 1. Then the utility of player 1 can obtain from this strategy is not more than

$$\max\{\delta (y_{1}^{*}+\lambda_{1}(y_{1}^{*}-r_{1})),\delta^{2}(x_{1}^{*}+\lambda_{1}(x_{1}^{*}-r_{1}))\}$$

Thus, *z* is not optimal.

If *t* is even, then player 1 has to respond to the proposal *z* made by player 2. As mentioned above, it is optimal for player 1 to follow strategy $\hat{f}$ at time *t* + 1. Therefore, it follows that accepting *z* is optimal for player 1 if and only if

$$z_{1}+\lambda_{1}(z_{1}-r_{1})>\delta (x_{1}^{*}+\lambda_{1}(x_{1}^{*}-r_{1}))$$

Thus, we have proved that player 1 cannot improve the share of the pie by deviating from strategy $\hat{f}$ at any single time *t*, given the strategy $\hat{g}$ for player 2.

Similarly, we can prove that player 2 cannot improve the share of the pie by unilaterally changing strategy $\hat{g}$ at any single time *t*, given the strategy $\hat{f}$ for player 1. □

Obviously, if *λ*_1_ = *λ*_2_ = 0, then the equilibrium partition is identical to the classical Rubinstein proposals, i.e.,

(1/(1+δ),δ/(1+δ))

If fairness concern parameters are equal and satisfy*λ* = *λ*_1_ = *λ*_2_ > 0, then the equilibrium partition of the bargaining game is

$$x^{*}=(\frac{1}{1+\delta }+\frac{(\alpha -\frac{1}{1+\delta })\lambda }{1+\lambda },\frac{\delta }{1+\delta }-\frac{(\alpha -\frac{1}{1+\delta })\lambda }{1+\lambda }).$$

An interesting observation is that player 1, who starts making an offer, has an advantage compared to the Rubinstein case if *α* > 1/(1+δ), where 1/(1+δ) reflects the bargaining power of player 1 in the classical Rubinstein bargaining game [3]. In this case, if *α* > 1/(1+δ), then

$$\frac{1}{1+\delta }+\frac{(\alpha -\frac{1}{1+\delta })\lambda }{1+\lambda }>\frac{1}{1+\delta },$$

which implies that player 1 benefits from fairness concerns. Another observation is that player 1 is hurt by fairness concerns if *α* < 1/(1+δ).

It is worthwhile noting that the equilibrium payoff is identical to the classical Rubinstein proposals when *α* is equal to 1/(1+δ), which means that players’ fairness concerns have no effect on the outcome of the bargaining game.

To show the uniqueness of the strategy profile ($\hat{f},\hat{g}$), the set of player *i*‘s SPE payoffs is denoted by *Λ_i_* in any subgame where player *i* first makes an offer. Let *b* = (*b*_1_, *b*_2_) be denoted as a SPE partition in any subgame, where player *i* first makes an offer. Formally, *Λ_i_* = {*b_i_*}. For any subgame where player *i* first makes an offer, *M_i_* denotes the supremum of *Λ_i_* and *m_i_* denotes the infimum of *Λ_i_*.

**Lemma** **1.** *For the alternating-offers bargaining model with fairness concerns, m_1_, m_2_, M_1_ and M_2_ satisfy the following conditions:*

$$m_{1}\geq 1-\delta M_{2}-\left[(1-\delta )\lambda_{2}r_{2}\right]/\left(1+\lambda_{2}\right)\;\mathrm{and}\;m_{2}\geq 1-\delta M_{1}-\left[(1-\delta )\lambda_{1}r_{1}\right]/\left(1+\lambda_{1}\right).$$

**Proof.**  In any SPE, player 2 accepts any offer *x* such that

$$x_{2}+\lambda_{2}(x_{2}-r_{2})>\delta (M_{2}+\lambda_{2}(M_{2}-r_{2}))$$

or

$$(1-x_{1})+\lambda_{2}((1-x_{1})-r_{2})>\delta (M_{2}+\lambda_{2}(M_{2}-r_{2})),$$

i.e.,

$$1-\delta M_{2}-\left[(1-\delta )\lambda_{2}r_{2}\right]/\left(1+\lambda_{2}\right)>x_{1}.$$

Thus, there does not exist *b*_1_ ∈ *Λ*_1_ such that $b_{1}<1-\delta M_{2}-\left[(1-\delta )\lambda_{2}r_{2}\right]/\left(1+\lambda_{2}\right)$. Otherwise, another proposal x^=(x1^,x2^) is made such that

b1<x1^<1−δM2−[(1−δ)λ2r2]/(1+λ2)

to increase the payoff of player 1. Hence, for any *b*_1_ ∈ *Λ*_1_, we have $b_{1}\geq 1-\delta M_{2}-$$\left[(1-\delta )\lambda_{2}r_{2}\right]/\left(1+\lambda_{2}\right)$, which implies that

$$m_{1}\geq 1-\delta M_{2}-\left[(1-\delta )\lambda_{2}r_{2}\right]/\left(1+\lambda_{2}\right).$$

Similarly, we can prove $m_{2}\geq 1-\delta M_{1}-\left[(1-\delta )\lambda_{1}r_{1}\right]/\left(1+\lambda_{1}\right)$.

These complete the proof of Lemma 1. □

**Lemma** **2.** *For the alternating-offers bargaining model with fairness concerns*

*(i)*  *for any b_2_∈Λ_2_,* $1-\delta b_{2}-\left[(1-\delta )\lambda_{2}r_{2}\right]/\left(1+\lambda_{2}\right)$ *∈ Λ_1_*
*(ii)*  *for any b_1_∈Λ_1_,* $1-\delta b_{1}-\left[(1-\delta )\lambda_{1}r_{1}\right]/\left(1+\lambda_{1}\right)$ *∈ Λ_2_.*

**Proof.**  Given any *b*_2_∈*Λ*_2_, let the SPE *σ* support player 2′s share *b*_2_, then player 1′s payoff in this equilibrium be denoted by *b*_1_. For any subgame where player 1 first makes an offer, the following strategies are considered. Player 1 first makes the offer x^=(x1^,x2^) such that

x2^+λ2(x2^−r2)=δ(b2+λ2(b2−r2)),

or,

1−x1^+λ2((1−x1^)−r2)=δ(b2+λ2(b2−r2))

x1^=1−δb2−[(1−δ)λ2r2]/(1+λ2).

and player 2 accepts a proposal *x* = (*x*_1_, *x*_2_) if and only if $x_{2}+\lambda_{2}(x_{2}-r_{2})$ ≥ x2^+λ2(x2^−r2). If any proposal is rejected, then the bargaining game continues according to *σ*. Thus, this pair of strategies is a SPE. Since $b_{1}+b_{2}=1,b_{1}\geq \delta b_{1}+\left[(1-\delta )\lambda_{1}r_{1}\right]/\left(1+\lambda_{1}\right)$ and $b_{2}\geq \delta b_{2}+\left[(1-\delta )\lambda_{2}r_{2}\right]/\left(1+\lambda_{2}\right)$, we have

$$\delta b_{1}+\left[(1-\delta )\lambda_{1}r_{1}\right]/\left(1+\lambda_{1}\right)+\delta b_{2}+\left[(1-\delta )\lambda_{2}r_{2}\right]/\left(1+\lambda_{2}\right)\leq 1.$$

Thus,

δb1+[(1−δ)λ1r1]/(1+λ1)≤1−δb2−[(1−δ)λ2r2]/(1+λ2)=x1^.

Thus, we have

x1^+λ1(x1^−r1)≥δ(b1+λ1(b1−r1)).

That is, player 1 cannot benefit from an offer that player 1′s payoff is greater than x1^. Thus, we have

x1^=1−δb2−[(1−δ)λ2r2]/(1+λ2)∈Λ1.

Similarly, we can prove $1-\delta b_{1}-\left[(1-\delta )\lambda_{1}r_{1}\right]/\left(1+\lambda_{1}\right)$ ∈ *Λ*_2_.

These complete the proof of Lemma 2. □

**Lemma** **3.** *For the alternating-offers bargaining model with fairness concerns, m_1_, m_2_, M_1_ and M_2_ satisfy the following conditions:*

*(i)* 
$m_{1}\leq 1-\delta M_{2}-\left[(1-\delta )\lambda_{2}r_{2}\right]/\left(1+\lambda_{2}\right)$ *and* $M_{1}\geq 1-\delta m_{2}-\left[(1-\delta )\lambda_{2}r_{2}\right]/\left(1+\lambda_{2}\right)$ *;*
*(ii)* 
$m_{2}\leq 1-\delta M_{1}-\left[(1-\delta )\lambda_{1}r_{1}\right]/\left(1+\lambda_{1}\right)$ *and* $M_{2}\geq 1-\delta m_{1}-\left[(1-\delta )\lambda_{1}r_{1}\right]/\left(1+\lambda_{1}\right)$ *.*

**Proof.**  If $m_{1}>1-\delta M_{2}-\left[(1-\delta )\lambda_{2}r_{2}\right]/\left(1+\lambda_{2}\right)$, then there exists *b*_2_ ∈ *Λ*_2_ such that $m_{1}>1-\delta b_{2}-\left[(1-\delta )\lambda_{2}r_{2}\right]/\left(1+\lambda_{2}\right)$. This contradicts $1-\delta b_{2}-\left[(1-\delta )\lambda_{2}r_{2}\right]/\left(1+\lambda_{2}\right)$ ∈ *Λ*_1_.

Similarly, if $M_{1}<1-\delta m_{2}-\left[(1-\delta )\lambda_{2}r_{2}\right]/\left(1+\lambda_{2}\right)$, then there exists *b*_2_∈*Λ*_2_ such that

$M_{1}<1-\delta b_{2}-\left[(1-\delta )\lambda_{2}r_{2}\right]/\left(1+\lambda_{2}\right)$, which contradicts $1-\delta b_{2}-\left[(1-\delta )\lambda_{2}r_{2}\right]/\left(1+\lambda_{2}\right)$ ∈ *Λ*_1_.

Similarly, we can prove Lemma 3 (ii).

These complete the proof of Lemma 3. □

**Lemma** **4.** *For the alternating-offers bargaining model with fairness concerns,*

*(i)* 
$M_{1}\leq 1-\delta m_{2}-\left[(1-\delta )\lambda_{2}r_{2}\right]/\left(1+\lambda_{2}\right)$ *,*
*(ii)* 
$M_{2}\leq 1-\delta m_{1}-\left[(1-\delta )\lambda_{1}r_{1}\right]/\left(1+\lambda_{1}\right)$ *.*

**Proof.**  Given any subgame, where player 1 first makes an offer, there exists the set of SPE divided into the following two cases:

Case 1: in this equilibrium, the initial offer of player 1 *x* = (*x*_1_, *x*_2_) is accepted;

Case 2: in this equilibrium, the initial offer of player 1 *x* = (*x*_1_, *x*_2_) is rejected.

Note that player 2 rejects any proposal that satisfies

$$x_{2}+\lambda_{2}(x_{2}-r_{2})<\delta (m_{2}+\lambda_{2}(m_{2}-r_{2}))\;\mathrm{or}\;x_{2}<\delta m_{2}+\left[(1-\delta )\lambda_{2}r_{2}\right]/\left(1+\lambda_{2}\right)$$

in any SPE.

Thus, for case 1, the most share that player 1 obtains is

(7) $$1-\delta m_{2}-\left[(1-\delta )\lambda_{2}r_{2}\right]/\left(1+\lambda_{2}\right).$$

For case 2, the agreement is delayed. The outcome in any SPE has present value to player 1 no higher than

(8) $$\delta (1-\delta m_{2}-\left[(1-\delta )\lambda_{2}r_{2}\right]/\left(1+\lambda_{2}\right))<1-\delta m_{2}-\left[(1-\delta )\lambda_{2}r_{2}\right]/\left(1+\lambda_{2}\right)$$

Thus, we have

$$M_{1}\leq 1-\delta m_{2}-\left[(1-\delta )\lambda_{2}r_{2}\right]/\left(1+\lambda_{2}\right)$$

Similarly, the proof of Lemma 4 (ii) can be shown.

These complete the proof of Lemma 4. □

**Theorem** **3.** *For the alternating-offers bargaining model with fairness concerns, (* $\hat{f},\hat{g}$ *) is the unique SPE strategy profile, if it satisfies properties (i)−(ii).*

**Proof.**  It follows from Lemmas 1, 3 and 4 that

(9) $$M_{1}=1-\delta m_{2}-\left[(1-\delta )\lambda_{2}r_{2}\right]/\left(1+\lambda_{2}\right)$$

(10) $$m_{1}=1-\delta M_{2}-\left[(1-\delta )\lambda_{2}r_{2}\right]/\left(1+\lambda_{2}\right)$$

(11) $$m_{2}=1-\delta M_{1}-\left[(1-\delta )\lambda_{1}r_{1}\right]/\left(1+\lambda_{1}\right)$$

(12) $$M_{2}=1-\delta m_{1}-\left[(1-\delta )\lambda_{1}r_{1}\right]/\left(1+\lambda_{1}\right)$$

Combining Equations (9)−(12) and Theorem 1, we have

$$M_{1}=m_{1}=\frac{1}{1+\delta }-\frac{(1-\alpha )\lambda_{2}-\delta \alpha \lambda_{1}}{\left(1+\delta )(1+\alpha (\lambda_{1}-\lambda_{2})+\lambda_{2}\right)}$$

and

$$M_{2}=m_{2}=\frac{1}{1+\delta }-\frac{\alpha \lambda_{1}-(1-\alpha )\delta \lambda_{2}}{\left(1+\delta )(1+\alpha (\lambda_{1}-\lambda_{2})+\lambda_{2}\right)}.$$

Thus, player 1 always plays $\hat{f}$ and player 2 always plays $\hat{g}$.

Therefore, the strategy profile ($\hat{f},\hat{g}$) is the unique SPE strategy profile.

The proof of Theorem 3 is completed. □

Here, we discuss the effect of fairness concern coefficients *λ*_1_ and *λ*_2_ and player 1′s bargaining power *α* on the SPE.

Recall that

$$x^{*}=(\frac{1}{1+\delta }-\frac{(1-\alpha )\lambda_{2}-\delta \alpha \lambda_{1}}{\left(1+\delta )(1+\alpha (\lambda_{1}-\lambda_{2})+\lambda_{2}\right)},\frac{\delta }{1+\delta }+\frac{(1-\alpha )\lambda_{2}-\delta \alpha \lambda_{1}}{\left(1+\delta )(1+\alpha (\lambda_{1}-\lambda_{2})+\lambda_{2}\right)}).$$

Since what player 1 gains is exactly what player 2 loses, we restrict ourselves to player 1. Differentiating with respect to *λ*_1_, *λ*_2_, respectively, we have

$$\frac{dx_{1}^{*}}{d\lambda_{1}}=\frac{\alpha \delta +(1-\alpha )(1+\delta )\lambda_{2})}{(1+\delta ){\left(1+\alpha (\lambda_{1}-\lambda_{2})+\lambda_{2}\right)}^{2}}>0\;\mathrm{and}\;\frac{dx_{1}^{*}}{d\lambda_{2}}=-\frac{\left(1-\alpha )(1+\alpha (1+\delta )\lambda_{1}\right)}{(1+\delta ){\left(1+\alpha (\lambda_{1}-\lambda_{2})+\lambda_{2}\right)}^{2}}<0.$$

It is easy to know that player 1 benefits from fairness concern coefficient *λ*_1_ and is hurt by fairness concern coefficient *λ*_2_.

Similarly, differentiating with respect to *α*, we have

$$\frac{dx_{1}^{*}}{d\alpha }=\frac{\lambda_{2}+\delta_{2}\lambda_{1}+(1+\delta )\lambda_{1}\lambda_{2}}{(1+\delta ){\left(1+\alpha (\lambda_{1}-\lambda_{2})+\lambda_{2}\right)}^{2}}>0.$$

It is shown that player 1 can benefit from *α*, which means that, for player 1, higher bargaining power can lead to a higher equilibrium share.

## 4. Extended Model

To check the robustness of the developed model, the model is generalized to the situation that both players have individual discount factors δ1 and δ2. Following the prior works (e.g., [42,43]), let *β_i_* > 0 be a positive rate of time preference for player *i*, then *δ_i_* = exp(−*β_i_*Δ). Equations (5) and (6) generalize to

$$x_{2}^{*}+\lambda_{2}(x_{2}^{*}-r_{2})=\delta_{2}(y_{2}^{*}+\lambda_{2}(y_{2}^{*}-r_{2}))$$

and

$$y_{1}^{*}+\lambda_{1}(y_{1}^{*}-r_{1})=\delta_{1}(x_{1}^{*}+\lambda_{1}(x_{1}^{*}-r_{1}))$$

All prior results can also be applied to this more general model. A solution to the above-mentioned two equalities is obtained from which a strategy profile is constructed. The strategy profile is the unique SPE that satisfies properties (i)−(ii). The outcome is

$$\begin{array}{l} x^{*}=(\frac{1-\delta_{2}}{1-\delta_{1}\delta_{2}}-\frac{(1-\alpha )(1-\delta_{2})\lambda_{2}-\delta_{2}(1-\delta_{1})\alpha \lambda_{1}}{\left(1-\delta_{1}\delta_{2})(1+\alpha (\lambda_{1}-\lambda_{2})+\lambda_{2}\right)},\frac{\delta_{2}(1-\delta_{1})}{1-\delta_{1}\delta_{2}}+\frac{(1-\alpha )(1-\delta_{2})\lambda_{2}-\delta_{2}(1-\delta_{1})\alpha \lambda_{1}}{\left(1-\delta_{1}\delta_{2})(1+\alpha (\lambda_{1}-\lambda_{2})+\lambda_{2}\right)})\\ y^{*}=(\frac{\delta_{1}(1-\delta_{2})}{1-\delta_{1}\delta_{2}}+\frac{\alpha (1-\delta_{1})\lambda_{1}-(1-\alpha )\delta_{1}(1-\delta_{2})\lambda_{2}}{\left(1-\delta_{1}\delta_{2})(1+\alpha (\lambda_{1}-\lambda_{2})+\lambda_{2}\right)},\frac{1-\delta_{1}}{1-\delta_{1}\delta_{2}}-\frac{\alpha (1-\delta_{1})\lambda_{1}-(1-\alpha )\delta_{1}(1-\delta_{2})\lambda_{2}}{\left(1-\delta_{1}\delta_{2})(1+\alpha (\lambda_{1}-\lambda_{2})+\lambda_{2}\right)}). \end{array}$$

**Corollary** **1.** *In the alternating-offers bargaining game with fairness concerns, if the time interval between two consecutive offers tends to zero, i.e., Δ→0, the outcome of the game converges to*

$$(\frac{\beta_{2}}{\beta_{1}+\beta_{2}}-\frac{(1-\alpha )\beta_{2}\lambda_{2}-\beta_{1}\alpha \lambda_{1}}{\left(\beta_{1}+\beta_{2})(1+\alpha (\lambda_{1}-\lambda_{2})+\lambda_{2}\right)},\frac{\beta_{1}}{\beta_{1}+\beta_{2}}+\frac{(1-\alpha )\beta_{2}\lambda_{2}-\beta_{1}\alpha \lambda_{1}}{\left(\beta_{1}+\beta_{2})(1+\alpha (\lambda_{1}-\lambda_{2})+\lambda_{2}\right)}).$$

**Proof.** 
$$\begin{array}{l} \underset{\Delta \to 0}{\lim}x^{*}=(\underset{\Delta \to 0}{\lim}(\frac{1-\delta_{2}}{1-\delta_{1}\delta_{2}}-\frac{(1-\alpha )(1-\delta_{2})\lambda_{2}-\delta_{2}(1-\delta_{1})\alpha \lambda_{1}}{\left(1-\delta_{1}\delta_{2})(1+\alpha (\lambda_{1}-\lambda_{2})+\lambda_{2}\right)}),\underset{\Delta \to 0}{\lim}(\frac{\delta_{2}(1-\delta_{1})}{1-\delta_{1}\delta_{2}}+\frac{(1-\alpha )(1-\delta_{2})\lambda_{2}-\delta_{2}(1-\delta_{1})\alpha \lambda_{1}}{\left(1-\delta_{1}\delta_{2})(1+\alpha (\lambda_{1}-\lambda_{2})+\lambda_{2}\right)}))\\ \;\;\;=(\underset{\Delta \to 0}{\lim}(\frac{1-\exp(\beta_{2}\Delta )}{1-\exp(\beta_{1}\Delta +\beta_{2}\Delta )}-\frac{(1-\alpha )(1-\exp(\beta_{2}\Delta ))\lambda_{2}-\exp(\beta_{2}\Delta )(1-\exp(\beta_{1}\Delta ))\alpha \lambda_{1}}{\left(1-\exp(\beta_{1}\Delta +\beta_{2}\Delta ))(1+\alpha (\lambda_{1}-\lambda_{2})+\lambda_{2}\right)}),\\ \;\;\;\underset{\Delta \to 0}{\lim}(\frac{\exp(\beta_{2}\Delta )(1-\exp(\beta_{1}\Delta ))}{1-\exp(\beta_{1}\Delta +\beta_{2}\Delta )}+\frac{(1-\alpha )(1-\exp(\beta_{2}\Delta ))\lambda_{2}-\exp(\beta_{2}\Delta )(1-\exp(\beta_{1}\Delta ))\alpha \lambda_{1}}{\left(1-\exp(\beta_{1}\Delta +\beta_{2}\Delta ))(1+\alpha (\lambda_{1}-\lambda_{2})+\lambda_{2}\right)}))\\ \;\;\;=(\underset{\Delta \to 0}{\lim}(\frac{\beta_{2}\Delta }{(\beta_{1}+\beta_{2})\Delta }-\frac{(1-\alpha )\beta_{2}\Delta \lambda_{2}}{\left(\beta_{1}+\beta_{2})\Delta (1+\alpha (\lambda_{1}-\lambda_{2})+\lambda_{2}\right)}+\frac{\beta_{1}\Delta \alpha \lambda_{1}}{\left(\beta_{1}+\beta_{2})\Delta (1+\alpha (\lambda_{1}-\lambda_{2})+\lambda_{2}\right)}),\\ \;\;\;\underset{\Delta \to 0}{\lim}(\frac{\beta_{1}\Delta }{(\beta_{1}+\beta_{2})\Delta }+\frac{(1-\alpha )\beta_{2}\Delta \lambda_{2}}{\left(\beta_{1}+\beta_{2})\Delta (1+\alpha (\lambda_{1}-\lambda_{2})+\lambda_{2}\right)}-\frac{\beta_{1}\Delta \alpha \lambda_{1}}{\left(\beta_{1}+\beta_{2})\Delta (1+\alpha (\lambda_{1}-\lambda_{2})+\lambda_{2}\right)}))\\ \;\;\;=(\frac{\beta_{2}}{\beta_{1}+\beta_{2}}-\frac{(1-\alpha )\beta_{2}\lambda_{2}-\beta_{1}\alpha \lambda_{1}}{\left(\beta_{1}+\beta_{2})(1+\alpha (\lambda_{1}-\lambda_{2})+\lambda_{2}\right)},\frac{\beta_{1}}{\beta_{1}+\beta_{2}}+\frac{(1-\alpha )\beta_{2}\lambda_{2}-\beta_{1}\alpha \lambda_{1}}{\left(\beta_{1}+\beta_{2})(1+\alpha (\lambda_{1}-\lambda_{2})+\lambda_{2}\right)})=\underset{\Delta \to 0}{\lim}y^{*}. \end{array}$$

These complete the proof of Corollary 1. □

Note that the equilibrium partition for Δ→0 is critically influenced by the players’ discount rates. The player’s equilibrium payoff depends on the ratio *β*_1_*/β*_2_.

## 5. An Application to Bilateral Monopoly Market

The above sections refer to the alternating-offer bargaining model between two players. From now on, we focus on the application of the proposed bargaining model in a market with a single buyer and a single seller. To illustrate the validity of our bargaining model, the professional basketball market in North America is considered. In the professional basketball market, the National Basketball Association (NBA) monopolizes professional basketball games in America, while the National Basketball Players Association (NBPA) monopolizes the professional player market. Thus, the NBA can only employ professional basketball players from the NBPA, that is, the professional basketball market can be regarded as a bilateral monopoly market with a single buyer (i.e., the NBA) and a single seller (i.e., the NBPA). In this market, the NBA and the NBPA bargain over players’ salaries and quantity. If an agreement cannot be reached, then the NBA seasons, including the NBA regular season, the NBA preceding season, the NBA playoffs and the NBA finals, may be delayed or canceled.

The NBA can employ *q* professional basketball players from the NBPA and benefit from these professional basketball players by playing basketball in the NBA season. Let *R*(*q*) denote by the revenue that is obtained by the NBA, and *R*(0) = 0, *R*′(*q*) > 0 and *R*″(*q*) < 0. Let *C*(*q*) be the NBPA ’s cost of provide *q* professional basketball players, and *C*(0) = 0,*C*′(*q*) > 0 and *C*″(*q*) > 0. Let *R*′(0) > *C*′(0). If the NBA decides to employ *q* professional basketball playersat a unit price *p*, then the NBA’s payoff function is

(13) $$\pi_{b}(p,q)=R(q)-pq,$$

and the NBPA ’s payoff function is

(14) $$\pi_{s}(p,q)=pq-C(q).$$

Thus, the total surplus is

(15) π(q)=R(q)−C(q).

The NBA and the NBPA bargain over the quantity *q* and the salary *p* according to the alternating-offers procedure. The bargaining begins with an offer by the NBA. It is assumed that the NBA and the NBPA pay attention to fairness. Let *α* be denoted by the NBA’s bargaining power, then 1−*α* reflects the NBPA’s bargaining power. The fairness reference is the Nash bargaining solution. Let *r_b_* and *r_s_* denote by the fairness reference points of the NBA and the NBPA, respectively. It follows from Theorem 1 that

$$r_{b}=\frac{\alpha (1+\lambda_{1})}{1+\alpha (\lambda_{1}-\lambda_{2})+\lambda_{2}}\pi (q)\;\mathrm{and}\;r_{s}=\frac{\left(1-\alpha )(1+\lambda_{2}\right)}{1+\alpha (\lambda_{1}-\lambda_{2})+\lambda_{2}}\pi (q),$$

respectively. An offer is denoted by (*p*, *q*), where *p* ≥ 0 and *q* ≥ 0. If the two players reach an agreement on (*p*, *q*) at time *t*, then the NBA’s utility function is

(16) $$u_{b}(p,q)=[\pi_{b}(p,q)+\lambda_{b}(\pi_{b}(p,q)-\frac{\alpha (1+\lambda_{b})}{1+\alpha (\lambda_{b}-\lambda_{s})+\lambda_{s}}\pi (q))]\exp(-\beta \Delta ),$$

and the NBPA ’s utility function is

(17) $$u_{s}(p,q)=[\pi_{s}(p,q)+\lambda_{s}(\pi_{s}(p,q)-\frac{\left(1-\alpha )(1+\lambda_{s}\right)}{1+\alpha (\lambda_{b}-\lambda_{s})+\lambda_{s}}\pi (q))]\exp(-\beta \Delta ),$$

On the other hand, if the agreement cannot be reached forever, then the utility of each player is zero.

### 5.1. SPE in Bargaining Game with the NBA and the NBPA

Let ($p_{b}^{*},q_{b}^{*}$) be the equilibrium offer that the NBA makes and ($p_{s}^{*},q_{s}^{*}$) be the equilibrium offer that the NBPA makes. According to the property, the NBPA ’s offer should make the NBA indifferent between this offer and the NBA’s own offer in the next phase. Thus, we have

$$u_{b}(p_{s}^{*},q_{s}^{*})=\delta u_{b}(p_{b}^{*},q_{b}^{*})$$

i.e.,

(18) $$\begin{array}{l} \pi_{b}(p_{s}^{*},q_{s}^{*})+\lambda_{b}(\pi_{b}(p_{s}^{*},q_{s}^{*})-\frac{\alpha (1+\lambda_{b})}{1+\alpha (\lambda_{b}-\lambda_{s})+\lambda_{s}}\pi (q_{s}^{*}))\\ =\delta [\pi_{b}(p_{b}^{*},q_{b}^{*})+\lambda_{b}(\pi_{b}(p_{b}^{*},q_{b}^{*})-\frac{\alpha (1+\lambda_{b})}{1+\alpha (\lambda_{b}-\lambda_{s})+\lambda_{s}}\pi (q_{b}^{*}))] \end{array}$$

($p_{s}^{*},q_{s}^{*}$) of the NBPA maximizes the utility $u_{s}(p_{s},q_{s})$ subject to

$$u_{b}(p_{s},q_{s})=\delta u_{b}(p_{b}^{*},q_{b}^{*}).$$

It means that

(19) $$q_{s}^{*}=q_{e},$$

where *q_e_* is the unique solution to

$$\frac{\partial \pi (q)}{\partial q}=R^{\prime }(q)-C^{\prime }(q)=0.$$

Since $\pi_{b}(p_{s}^{*},q_{s}^{*})+\pi_{s}(p_{s}^{*},q_{s}^{*})=\pi (q_{e})$, where

(20) $$\pi (q_{e})=R(q_{e})-C(q_{e}).$$

Combining Equations (19) and (20), we have

(21) $$\begin{array}{l} \pi (q_{e}^{})-\pi_{s}(p_{s}^{*},q_{e}^{})+\lambda_{b}(\pi (q_{e}^{})-\pi_{s}(p_{s}^{*},q_{e}^{})-\frac{\alpha (1+\lambda_{b})}{1+\alpha (\lambda_{b}-\lambda_{s})+\lambda_{s}}\pi (q_{e}^{}))\\ =\delta [\pi_{b}(p_{b}^{*},q_{b}^{*})+\lambda_{b}(\pi_{b}(p_{b}^{*},q_{b}^{*})-\frac{\alpha (1+\lambda_{b})}{1+\alpha (\lambda_{b}-\lambda_{s})+\lambda_{s}}\pi (q_{b}^{*}))] \end{array}$$

Similarly, we have

$$u_{s}(p_{b}^{*},q_{b}^{*})=\delta u_{s}(p_{s}^{*},q_{s}^{*})$$

i.e.,

(22) $$\begin{array}{l} \pi_{s}(p_{b}^{*},q_{b}^{*})+\lambda_{s}(\pi_{s}(p_{b}^{*},q_{b}^{*})-\frac{\left(1-\alpha )(1+\lambda_{s}\right)}{1+\alpha (\lambda_{b}-\lambda_{s})+\lambda_{s}}\pi (q_{b}^{*}))\\ =\delta [\pi_{s}(p_{s}^{*},q_{s}^{*})+\lambda_{s}(\pi_{s}(p_{s}^{*},q_{s}^{*})-\frac{\left(1-\alpha )(1+\lambda_{s}\right)}{1+\alpha (\lambda_{b}-\lambda_{s})+\lambda_{s}}\pi (q_{s}^{*}))] \end{array}$$

($p_{b}^{*},q_{b}^{*}$) of the NBA maximizes his utility $u_{b}(p_{b},q_{b})$ subject to

$$u_{s}(p_{b},q_{b})=\delta u_{s}(p_{s}^{*},q_{s}^{*}).$$

This means that

(23) $$q_{b}^{*}=q_{e}.$$

Since $\pi_{b}(p_{b}^{*},q_{b}^{*})+\pi_{s}(p_{b}^{*},q_{b}^{*})=\pi (q_{e})$, combining Equations (22) and (23), we have

(24) $$\begin{array}{l} \pi (q_{e}^{})-\pi_{b}(p_{b}^{*},q_{e}^{})+\lambda_{s}(\pi (q_{e}^{})-\pi_{b}(p_{b}^{*},q_{e}^{})-\frac{\left(1-\alpha )(1+\lambda_{s}\right)}{1+\alpha (\lambda_{b}-\lambda_{s})+\lambda_{s}}\pi (q_{e}^{}))\\ =\delta [\pi_{s}(p_{s}^{*},q_{s}^{*})+\lambda_{s}(\pi_{s}(p_{s}^{*},q_{s}^{*})-\frac{\left(1-\alpha )(1+\lambda_{s}\right)}{1+\alpha (\lambda_{b}-\lambda_{s})+\lambda_{s}}\pi (q_{b}^{*}))] \end{array}$$

Combining Equations (20)–(24), we can obtain

(25) $$\pi_{b}(p_{b}^{*},q_{e})=\frac{\pi (q_{e})}{1+\delta }-\frac{(1-\alpha )\lambda_{s}-\delta \alpha \lambda_{b}}{\left(1+\delta )(1+\alpha (\lambda_{b}-\lambda_{s})+\lambda_{s}\right)}\pi (q_{e})$$

and

(26) $$\pi_{s}(p_{s}^{*},q_{s})=\frac{\pi (q_{e})}{1+\delta }-\frac{\alpha \lambda_{b}-\delta (1-\alpha )\lambda_{s}}{\left(1+\delta )(1+\alpha (\lambda_{b}-\lambda_{s})+\lambda_{s}\right)}\pi (q_{e}).$$

It follows from Equations (13), (15), (20) and (25) that a player’s salary is

(27) $$p_{b}^{*}=(\frac{\delta }{1+\delta }+\frac{(1-\alpha )\lambda_{s}-\delta \alpha \lambda_{b}}{\left(1+\delta )(1+\alpha (\lambda_{b}-\lambda_{s})+\lambda_{s}\right)})\frac{R(q_{e})}{q_{e}}+(\frac{1}{1+\delta }-\frac{(1-\alpha )\lambda_{s}-\delta \alpha \lambda_{b}}{\left(1+\delta )(1+\alpha (\lambda_{b}-\lambda_{s})+\lambda_{s}\right)})\frac{C(q_{e})}{q_{e}}.$$

Similarly, it follows from Equations (14), (15), (19) and (26) that a player’s salary is

(28) $$p_{s}^{*}=(\frac{1}{1+\delta }-\frac{\alpha \lambda_{b}-\delta (1-\alpha )\lambda_{s}}{\left(1+\delta )(1+\alpha (\lambda_{b}-\lambda_{s})+\lambda_{s}\right)})\frac{R(q_{e})}{q_{e}}+(\frac{\delta }{1+\delta }+\frac{\alpha \lambda_{b}-\delta (1-\alpha )\lambda_{s}}{\left(1+\delta )(1+\alpha (\lambda_{b}-\lambda_{s})+\lambda_{s}\right)})\frac{C(q_{e})}{q_{e}}.$$

By straightforwardly adapting the proof of Theorem 2, it is shown that ($p_{b}^{*},q_{e}$) is a SPE strategy profile. We can also prove that ($p_{b}^{*},q_{e}$) is the unique SPE in a bilateral monopoly game by straightforwardly adapting agreements of Lemmas 1–4. That is, $p_{b}^{*}$ is the salary that the NBA pays to a player.

### 5.2. Properties of the Unique SPE in Bargaining Games between the NBA and the NBPA

In this subsection, we analyze the equilibrium price with respect to the fairness concern coefficients *λ*_1_ and *λ*_2_ and the NBA’s bargaining power *α*.

Recall the unique SPE ($p_{b}^{*},q_{e}$), where

$$p_{b}^{*}=(\frac{\delta }{1+\delta }+\frac{(1-\alpha )\lambda_{s}-\delta \alpha \lambda_{b}}{\left(1+\delta )(1+\alpha (\lambda_{b}-\lambda_{s})+\lambda_{s}\right)})\frac{R(q_{e})}{q_{e}}+(\frac{1}{1+\delta }-\frac{(1-\alpha )\lambda_{s}-\delta \alpha \lambda_{b}}{\left(1+\delta )(1+\alpha (\lambda_{b}-\lambda_{s})+\lambda_{s}\right)})\frac{C(q_{e})}{q_{e}}.$$

Since

$$\frac{dp_{b}^{*}}{d\lambda_{b}}=-\frac{\alpha (\delta +(1-\alpha )(1+\delta )\lambda_{b})}{(1+\delta ){\left(1+\alpha (\lambda_{b}-\lambda_{s})+\lambda_{s}\right)}^{2}}\frac{R(q_{e})-C(q_{e})}{q_{e}}<0$$

and

$$\frac{dp_{b}^{*}}{d\lambda_{s}}=\frac{\left(1-\alpha )(1+\alpha (1+\delta )\lambda_{b}\right)}{(1+\delta ){\left(1+\alpha (\lambda_{b}-\lambda_{s})+\lambda_{s}\right)}^{2}}\frac{R(q_{e})-C(q_{e})}{q_{e}}>0,$$

The more the NBA concerns fairness, the lower the equilibrium price is. The more the NBPA concerns fairness, the higher the equilibrium price is.

Differentiating with respect to *α* yields

$$\frac{dp_{b}^{*}}{d\alpha }=-\frac{\delta \lambda_{b}\lambda_{s}+\delta \lambda_{b}+\lambda_{b}\lambda_{s}+\lambda_{s}}{(1+\delta ){\left(1+\alpha (\lambda_{b}-\lambda_{s})+\lambda_{s}\right)}^{2}}\frac{R(q_{e})-C(q_{e})}{q_{e}}<0.$$

Therefore, a high bargaining power for the NBA leads to a low salary for NBA players.

Note that if *λ_b_* = *λ_s_* = 0, then the outcome collapses to that of Muthoo [2], i.e., $p_{M}^{*}=\frac{\delta }{1+\delta }\frac{R(q_{e})}{q_{e}}+\frac{1}{1+\delta }\frac{C(q_{e})}{q_{e}}.$

### 5.3. Numerical Analysis

**Figure 1:**
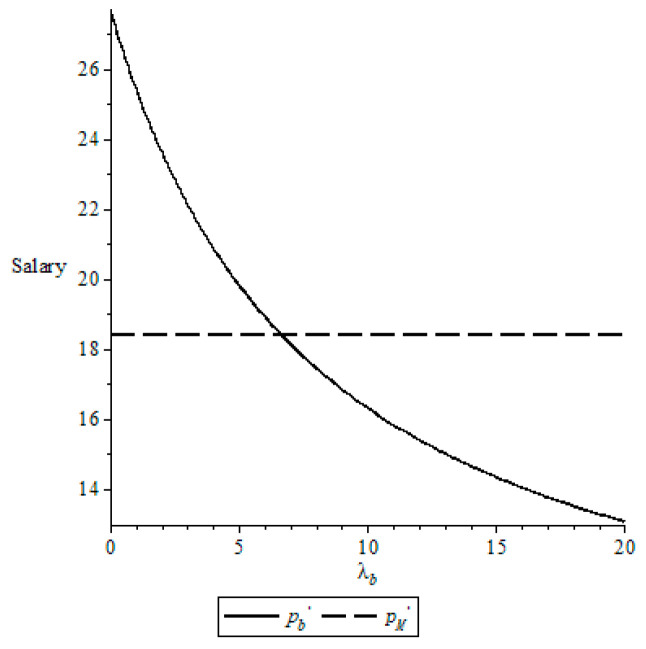
Change of the salary with *λ_b_*.

The NBA possesses 30 teams, each of which consists of 17 active players (https://china.nba.cn/playerindex/, accessed on 21 January 2023). That is, the NBA possesses 510 active players. Adam Silver, who is the NBA commissioner, reported that the total revenue of the NBA is about $ 10 billion in the 2021–2022 season (https://rmh.pdnews.cn/Pc/ArtInfoApi/video?id=30010572, accessed on 18 July 2022). Consider the case that the number of players (who play basketball) for each team is at most 15 in the NBA season. This implies that the average revenue for active players is approximately equal to $ 27.8 million, that is, $R(q_{e})/q_{e}$ = $ 27.8 million. It is worthwhile noting that the operation cost of each team for the NBA is about $ 0.2 billion per year (http://www.guangdonglong.com/c/0G5HA2H022.html, accessed on 21 July 2022). This implies that the average operation cost for each active player is $ 11.8 million, i.e., $C(q_{e})/q_{e}$ = $ 11.8 million. Let *δ* = 0.7, *α* = 0.3 and *λ_s_* = 2, we examine the impact of fairness concern parameters *λ_b_* on the salary, as shown in Figure 1.

From Figure 1, a high fairness concern coefficient for the NBA leads to a small salary for each active player. That is, the NBA cares more about fairness, which causes it pays less salary to an active player. On the other hand, compared with the salary $p_{M}^{*}$ when the NBA and the NBPA are rational, there exists a threshold for the fairness concern coefficient for the NBA such that the salary $p_{b}^{*}$ for an active player satisfies $p_{b}^{*}>p_{M}^{*}$ if the fairness concern coefficient for the NBA is smaller than this threshold and $p_{b}^{*}<p_{M}^{*}$ otherwise. This means that, compared with the case where the NBA and the NBPA are rational, an active player can benefit from the fairness concerns for the NBA if the fairness concern coefficient for the NBA is low and be hurt by it otherwise.

**Figure 2:**
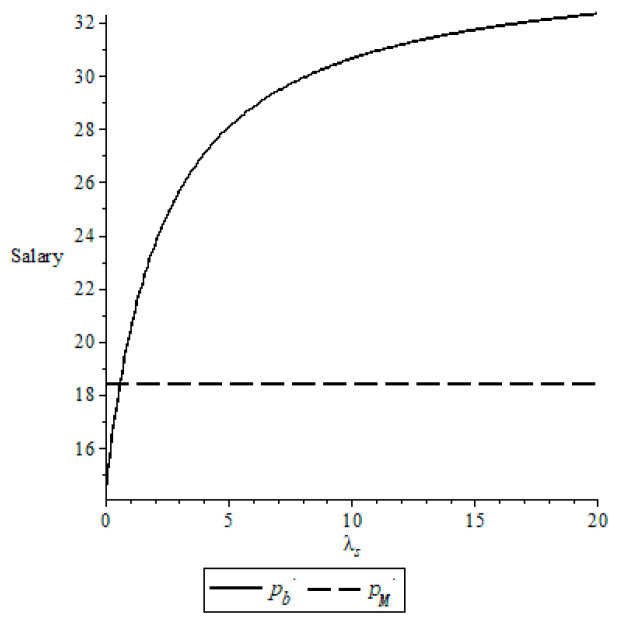
Change of the salary with *λ_s_*.

Let *δ* = 0.7, *α* = 0.3 and *λ_b_* = 2, we examine the impact of fairness concern parameters *λ_s_* on the salary, as shown in Figure 2.

From Figure 2, a high fairness concern coefficient for the NBA results in a high salary for an active player. That is, the NBPA cares more about fairness, which causes the NBA pays more salary to an active player. On the other hand, compared with the salary $p_{M}^{*}$ when the NBA and the NBPA are rational, there exists a threshold for the fairness concern coefficient for the NBPA such that $p_{b}^{*}<p_{M}^{*}$ if the fairness concern coefficient for the NBA is smaller than this threshold and $p_{b}^{*}>p_{M}^{*}$ otherwise. It means that, compared with the case where the NBA and the NBPA are rational, an active player is hurt by the fairness concerns of the NBPA if the fairness concern coefficient for the NBA is low and benefits from it otherwise.

**Figure 3:**
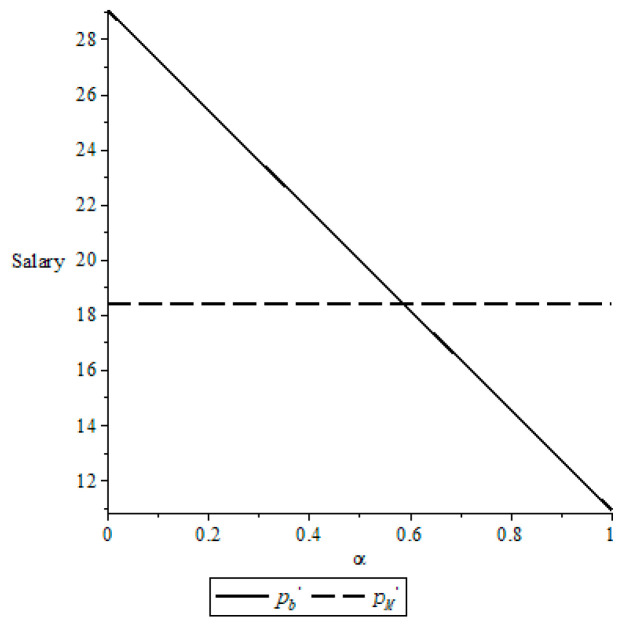
Change of the salary with *α*.

Let *δ* = 0.7 *λ_s_* = 2 and *λ_b_* = 2, we examine the impact of the bargaining power for the NBA *α* on the salary, as shown in Figure 3.

From Figure 3, a high bargaining power for the NBA leads to a low salary for an active player. That is, the higher the bargaining power for the NBA is, the less the salary for an active player is. In addition, compared with the salary $p_{M}^{*}$ when the NBA and the NBPA are rational, there exist a threshold for the bargaining power for the NBA such that $p_{b}^{*}>p_{M}^{*}$ if the bargaining power for the NBA is smaller than this threshold and $p_{b}^{*}<p_{M}^{*}$ otherwise. Therefore, compared with the case where the NBA and the NBPA are rational, an active player is hurt by low bargaining power for the NBA, and benefits from high bargaining power for the NBPA.

## 6. Discussion

From a theoretical perspective, the existing alternating-offer bargaining with fairness concerns has explored “Why are players willing to pay for fair treatment in some situations?” [35,36]. These studies consider the exogenous fairness reference without bargaining power, examining how players with inequality aversions split “a pie” equally. Such fairness reference level cannot exactly characterize the endogenous power of players such that inequality aversion perception is influenced. To address this issue, our work models the Rubinstein bargaining with fairness preferences under the assumption that the Nash bargaining solution is regarded as the fairness reference of each player. Compared to the alternating-offers bargaining game with rational players, a player (who starts making proposal) benefits from the fairness concerns if its bargaining power is high and it is hurt by the fairness concerns otherwise. 

Our results provide the following practical implications: (1) if a player cares more fairness, this player should make a high proposal when it is his turn to make proposals. (2) A player should make a low proposal if its opponent cares more about fairness. (3) A player with strong bargaining power should make a high proposal. To summarize, players should carefully weigh their own fairness concerns, bargaining power and fairness concerns of their opponents, and then make proposals, rather than simply follow the suggestion that the proposal at the current stage is higher than that at the past stages.

## 7. Conclusions and Limitations

In this paper, the effect of fairness concerns on the Rubinstein (1982) alternating-offers bargaining game is investigated. We construct a SPE in the alternating-offers bargaining game with fairness concerns and show its uniqueness. A sensitivity analysis on the equilibrium partition with respect to fairness concern coefficients is performed, we further find that the player’s equilibrium share of the pie is positively related to the fairness concerns of this player and negatively to the opponent’s fairness concern. It is shown that the payoff of each player depends on the ratio of discount rates of the two players if the time lapse between two offers goes to zero. Finally, an application to bilateral monopoly is given and the properties of equilibrium price are shown.

One limitation of this work is that players’ preferences are time-independent in Rubinstein’s bargaining game with fairness concerns. In numerous situations, this assumption is reasonable, while in others this assumption is often violated and players’ payoffs depend on the process of bargaining. Thus, the objective of future study is to discuss the effect of fairness concerns on the alternating-offers bargaining game with history-dependent preferences. In addition, the SPE is unique that shares two properties (i.e., No delay and Stationarity) with that of Rubinstein. It still is an open issue whether the uniqueness is also held without the above-mentioned two properties.

## Data Availability

All data, models, and code generated or used during the study appear in the submitted article.
